# The relationship between physical activity and mental health in middle-aged and older Chinese adults: a moderated mediation and cross-lagged model

**DOI:** 10.3389/fpsyg.2026.1773431

**Published:** 2026-03-02

**Authors:** Zhenhui Zhao, Xianliang Wang

**Affiliations:** School of Physical Education, Shandong University, Jinan, China

**Keywords:** cognitive function, mental health, middle aged, older adults, physical activity, sleep duration

## Abstract

**Background:**

Chronic psychological issues significantly exacerbate the risk of various diseases among middle-aged and older adults. Although prior research has indicated an association between physical activity and mental health, studies exploring the specific processes underlying this relationship remain insufficient. Therefore, this study aims to systematically investigate the relationship between physical activity and mental health in middle-aged and older populations, specifically by verifying the mediating role of sleep duration and the moderating role of cognitive function.

**Methods:**

Drawing on data from the China Health and Retirement Longitudinal Study (CHARLS) involving 12,656 middle-aged and older participants, we employed mediation and moderation analyses to explore variable associations. Furthermore, cross-lagged models were utilized to rigorously verify longitudinal causal effects over time.

**Results:**

Physical activity positively predicted the mental health (*β* = 0.002, *t* = −6.757, *p* < 0.001) and sleep duration (*β* = 0.001, *t* = 2.980, *p* < 0.01) of middle-aged and older adults. Sleep duration positively predicted mental health (*β* = 0.031, *t* = 32.825, *p* < 0.001), thereby verifying its mediating role. Meanwhile, cognitive function moderated the effect of physical activity on sleep duration (*β* = 0.001, *t* = −2.910, *p* < 0.01) and the effect of sleep duration on mental health (*β* = 0.009, *t* = 2.236, *p* < 0.05) in middle-aged and older adults. Regarding sub-dimensions, memory did not show a significant moderating effect. However, executive function significantly moderated the paths from physical activity to sleep duration (*β* = 0.001, *t* = −2.624, *p* < 0.01) and from sleep duration to mental health (*β* = 0.023, *t* = 1.775, *p* < 0.05). In the cross-lagged model, mental health positively predicted sleep duration (*β* = −0.129, *t* = −3.82, *p* < 0.001), and cognitive function moderated the impact of sleep duration on mental health (*β* = −0.062, *t* = −3.82, *p* < 0.05).

**Conclusion:**

(1) Physical activity is positively correlated with mental health in middle-aged and older adults; (2) sleep duration mediates the relationship between physical activity and mental health; (3) cognitive function plays a moderating role in both stages of the mediation process; (4) the executive function subdomain exhibits differential moderating effects in middle-aged and older adults compared to memory; (5) longitudinally, cognitive function predicts mental health and positively moderates the relationship between sleep duration and mental health.

## 1 Introduction

Mental health in middle-aged and older adults encompasses emotional well-being, stability, a positive outlook on life, and effective adaptation to one’s environment (McGorry et al., 2023). Robust mental health serves as the cornerstone of quality of life for this population; it not only enhances subjective well-being but also bolsters physical health, mitigates the risk of chronic diseases, and effectively extends healthy life expectancy. Currently, a significant number of middle-aged and older adults are plagued by mental health issues such as depression, stress, and anxiety. These psychological challenges are detrimental, often precipitating sleep disorders, appetite loss, fatigue, and somatic pain. Furthermore, chronic psychological distress increases susceptibility to cardiovascular disease, hypertension, and diabetes, thereby posing a severe threat to the longevity and quality of life of middle-aged and older adults (Yang et al., 2021). Consequently, conducting systematic and in-depth research into these mental health issues holds profound practical significance. Therefore, conducting systematic and in-depth research on mental health issues in middle-aged and older adults is of great practical significance.

With the intensification of global population aging, mental health issues among middle-aged and older adults have emerged as a core focus of academic research. As a non-pharmacological intervention, physical activity has garnered significant attention for the prevention and treatment of psychological problems, owing to its notable cost-effectiveness and safety profile. Existing research indicates that regular physical activity not only optimizes the physical and psychological health of older adults but also effectively alleviates depressive symptoms, thereby enhancing overall quality of life (Zhu et al., 2024). From a psychological perspective, mental health in middle-aged and older adults hinges on the satisfaction of basic needs and the regulation of self-acceptance; by reinforcing social interaction and self-efficacy, physical activity not only mitigates stress and anxiety and improves sleep quality but also elevates overall mental health levels (Biddle et al., 2019). From a physiological perspective, improvements in cerebral blood flow and the enhancement of neuroplasticity induced by physical activity, combined with the regulation of neuro-biomarker levels, collectively drive the functional remodeling of the central nervous system, thereby improving mental health status (Wang et al., 2025a).

Regarding the mechanisms linking physical activity to mental health, the majority of existing studies have employed cross-sectional designs. Some research has primarily focused on adolescents and university students: a cross-sectional survey indicated that physical activity positively predicts mental health in university students (Congsheng et al., 2022); another cross-sectional study targeting adolescents showed that physical activity is negatively correlated with psychological sub-health, implying that physical activity promotes mental health in this group (Shuaishuai, 2025). Other research focuses on the impact of different indicators. For instance, studies show that emotion regulation mediates the relationship between physical activity and health anxiety (Wang T. et al., 2025); a cross-sectional survey demonstrated that self-efficacy mediates the link between physical exercise and mental health, while emotion regulation and self-efficacy jointly mediate this relationship through a chain mediation effect (Hu and Liu, 2025).

However, despite middle-aged and older adults being a population with a high incidence of disease, relatively little is known about the impact of physical activity on their mental health. Furthermore, there is a significant gap in research that integrates key areas affecting mental health, such as sleep duration and cognitive function. Additionally, previous research models have mostly been limited to simple or chain mediation effects, often overlooking the examination of moderation mechanisms and longitudinal relationships between variables. Therefore, the innovation of this study lies in the construction of a moderated mediation model and a cross-lagged panel model. By positioning sleep duration as a mediator and cognitive function as a moderator, this study aims to systematically explore the multidimensional mechanisms of physical activity on the mental health of middle-aged and older adults from a comprehensive perspective, thereby providing references for promoting psychological well-being and reducing the risk of chronic diseases.

### 1.1 Theoretical framework

This study builds a theoretical framework based on activity theory, which posits that people engage in activities to achieve specific goals and can contribute to an individual’s mental health development (Dzewaltowski et al., 1990). In this study physical activity represents activity participation, sleep duration and cognitive functioning are body perceptions, and mental health responds to an individual’s internal psychological situation. This study is based on a suitable theoretical framework and introduces a wealth of theoretical support. For example, Positive Psychology guides the relationship between physical activity and mental health; Biological Clock Theory and Emotion Regulation Theory, which guide the mediating role of sleep duration between physical activity and mental health; and Cognitive Behavioral Theory, which guides the moderating role of cognitive functioning between physical activity, sleep duration, and mental health. These theories will be further explained in the hypotheses section of the study to enrich and refine the theoretical framework of this study.

### 1.2 Research hypothesis

#### 1.2.1 Physical activity and mental health

Physical activity mainly refers to the body activities caused by the contraction of skeletal muscles that are higher than the basal metabolic level of energy consumption, including such behaviors as work, household chores, leisure activities, sports and so on. Positive psychology suggests that physical activity effectively promotes the development of individual mental health by stimulating positive emotions, cognition, and behavior, making individuals more optimistic, confident, and hopeful for the future (Maurer and Daukantaitė, 2021). Positive Psychology A large number of studies have confirmed that there is a complex and profound relationship between physical activity and mental health. Physical activity can effectively improve the mood of individuals, reduce negative emotions such as anxiety, depression and stress, and promote the release of neurotransmitters such as endorphins and dopamine, so that individuals can produce pleasant emotions. A meta-analysis demonstrates that physical activity can induce a variety of physiological changes, enhancing an individual’s capacity to regulate stress and anxiety, thereby preventing psychological disorders and promoting overall well-being (Liu et al., 2025). Moreover, through scientific physical activity, individuals can significantly improve their physical fitness and body image, thereby enhancing self-confidence and self-esteem and improving their sense of self-identity. For example, through the investigation of Chinese obese and overweight college students, it was found that physical activity strengthened the subjects’ sense of self-identity and reduced their anxiety and depression (Wang et al., 2023). For middle-aged and older adults, the social component of structured physical activity plays a crucial role in alleviating loneliness. The primary mechanism underlying this effect is the enhancement of a sense of belonging and the reduction of perceived stress (Zhao et al., 2025). For example, in a longitudinal study of Chinese older adults, physical activity was found to improve social interaction and psychological well-being among older adults (Wang et al., 2019). The impact of physical activity on mental health is the effects of physical activity on mental health are multi-level and multi-dimensional, and through voluntary participation in appropriate physical activities, it can effectively promote the mental health level of individuals and enhance the quality of life. Furthermore, evidence suggests that physical activity, acting as a key factor regulating physical and mental health, facilitates the enhancement of self-efficacy and social engagement, thereby significantly improving life satisfaction and subjective well-being among older adults (Tao et al., 2025). Based on this, Hypothesis 1 is proposed.

*Hypothesis 1*: There is a positive relationship between physical activity and mental health in middle-aged and older adults.

#### 1.2.2 The mediating role of sleep duration

There is a close relationship between physical activity and sleep duration, which involves a variety of mechanisms, including physiology and psychology. Moderate physical activity is widely recognized as an effective non-pharmacological intervention to improve sleep efficiency, increase the duration of deep sleep, and reduce the number of nocturnal awakenings. The biological clock theory proposes that physical activity can regulate the body’s routine, allowing individuals to remain active during the day and feel more fatigued at night, thus improving their sleep quality. The biological clock theory proposes that physical activity regulates the body’s routine, allowing individuals to remain active during the day and feel more fatigued at night, thereby improving their sleep quality (Hozer and Pifferi, 2020). Specifically firstly, physical activity leads to an increase in an individual’s core temperature, followed by an effective sleep aid at the end of the exercise, during the process of decreasing and returning body temperature. For example, a related study investigated more than 1,600 residents in the community and found that physical activity affects the subjects’ body temperature and influences the quality of their sleep, and residents who are more physically active usually have better sleep state (Aoyagi et al., 2018). Secondly, physical activity can promote the secretion of endorphins and dopamine and other “happy hormones,” which can help to relieve the individual’s nervousness, thus improving the individual’s sleep quality. A meta-analysis indicates that physical activity influences the secretion and conversion of enzymes and hormones (e.g., by increasing melatonin secretion), thereby exerting a positive influence on sleep (Wang et al., 2025b). Finally, moderate physical activity can help individuals to adjust their circadian rhythms, improve their sleep structure, and increase the ratio of rapid eye movement (REM) to non-rapid eye movement (NREM) during sleep, which plays an important role in the recovery of the body and the rest of the brain. For example, in an 82-participant experiment, physical activity and REM were found to be related to sleep latency, and in particular, low-intensity and moderate-intensity physical activity were found to be effective in improving sleep quality in individuals (Zapalac et al., 2024). Therefore, incorporating moderate amounts of physical activity into an individual’s daily life can significantly improve sleep duration, thereby promoting physical and mental health and well-being.

Emotion regulation theory suggests that lack of sleep or poor quality of sleep will lead to greater fluctuations in mood, such as irritability, anxiety and depression, which is not conducive to the healthy development of the individual’s mental health (Lee et al., 2024). Specifically, adequate sleep is beneficial for emotional recovery and regulation; it facilitates the maintenance of emotional stability and positive affect, thereby promoting individual mental health. For example, in a study of 468 subjects, it was found that poor sleep quality directly enhances negative emotions such as anxiety, thus affecting their psychological health (Rakhimov et al., 2022). Moreover, sleep plays a crucial role in memory and cognitive function, and insufficient sleep can lead to issues such as memory loss and difficulty concentrating, thus affecting an individual’s mental health. For instance, a large-scale survey of European residents found that sleep quality has a significant impact on cognitive function, with the greatest effect observed in delayed recall (Cheval et al., 2022). Furthermore, adequate sleep is essential for immune function; it bolsters immunity and lowers susceptibility to disease, which in turn contributes to the preservation of mental well-being. For example, relevant studies have experimentally confirmed that insufficient sleep impairs both the number and function of immune cells. This weakens an individual’s immunity, potentially leading to disease and, consequently, affecting mental health (Bryant et al., 2004). Further research indicates that sleep regulates the efficiency of immune responses and inflammatory dynamics, playing a critical role in maintaining immune health. Conversely, chronic sleep deprivation significantly accelerates the progression of metabolic and neurodegenerative diseases (Singh et al., 2025). In conclusion, the impact of sleep duration on mental health involves various aspects such as emotional regulation, cognitive improvement and immune enhancement, and by adopting effective sleep management measures, the mental health of individuals can be significantly improved. Therefore, Hypothesis 2 of the study is proposed.

*Hypothesis 2*: Sleep duration mediates the link between physical activity and mental health in middle-aged and older adults.

#### 1.2.3 The moderating role of cognitive functions

Cognitive function refers to the brain’s ability to process, understand, apply and innovate information, which involves the human body perception, memory, thinking, language and other fields, and has an important impact on an individual’s mental health and learning ability, cognitive dysfunction can even lead to a serious decline in the quality of life and social functioning (Park et al., 2019). Cognitive-behavioral theory suggests that cognitive functioning plays a key role in individuals’ emotions and behaviors, and that individuals’ behavioral habits, such as physical activity and sleep, can influence their emotional performance and thus play a role in regulating their physical activity, sleep, and mental health (Scott et al., 2024).

Cognitive function plays a crucial role in the mechanism by which physical activity influences sleep duration. Physical activity increases physical fatigue, making it easier for individuals to fall asleep at night and enter deep sleep, thereby effectively extending sleep duration. Furthermore, individuals with higher cognitive function often engage in continuous mental processing during exercise. This sustained cognitive engagement leads to an increased consumption of cerebral metabolic resources, which exacerbates post-exercise fatigue and ultimately improves sleep duration. For instance, a survey of 325 employees found that excessive cognitive activity significantly increased fatigue, thereby indirectly affecting sleep (Varghese et al., 2020). On the other hand, circadian rhythms (the biological clock)—physiological and behavioral patterns with an approximate 24-h cycle—are a key factor influencing sleep duration. Research indicates that skeletal muscles communicate with the liver by secreting specific proteins to regulate circadian rhythms. In this context, regular physical activity is particularly important, as it not only maintains circadian stability but also improves sleep quality. For example, a study of 35 patients with Parkinson’s disease found that cognitive function was significantly correlated with circadian rhythm execution, as well as visuospatial and psychomotor components, thereby promoting sleep (Wu et al., 2018). Further research demonstrates that by activating the cognitive control functions of the prefrontal cortex, physical activity not only significantly enhances emotional regulation but also improves adherence to sleep schedules (Wang Y. et al., 2025).

Cognitive function plays a pivotal role in the mechanisms that regulate the impact of sleep duration on mental health. Adequate sleep duration helps to stabilize an individual’s mood, mitigates negative emotions such as anxiety and depression, and enhances overall psychological well-being. Cognitive function can improve mental health by processing external information positively, generating positive emotional experiences, and facilitating the maintenance of sufficient sleep duration. For instance, a study of 917 Chinese middle school students demonstrated that cognitive function effectively regulates emotions, thereby promoting psychological capital and life satisfaction (Xue et al., 2023). Additionally, sufficient sleep bolsters mental health by optimizing metabolism, regulating endocrine secretion, and strengthening immunity. Research on patients with severe obesity indicates that cognitive function influences hormone secretion, subsequently affecting metabolic and endocrine processes (Zhang et al., 2022). Through neuroendocrine regulation and the modulation of metabolic pathways, cognitive function can significantly impact metabolic levels and optimize sleep duration, ultimately promoting the mental health of individuals. Based on this, we propose Hypotheses 3, 4.

*Hypothesis 3*: Cognitive function plays a moderating effect between physical activity and sleep duration in middle-aged and older adults.

*Hypothesis 4*: Cognitive functioning in middle-aged and older adults plays a moderating effect between sleep duration and mental health.

#### 1.2.4 The moderating role of memory-executive

Episodic memory is a long-term declarative memory system involving the conscious recollection of personal events (Davies and Clayton, 2024). On the one hand, episodic memory plays a complex moderating role between physical activity and sleep duration through neurobiological and behavioral psychological mechanisms. Research indicates that physical activity enhances activation in specific brain regions of older adults, such as the entorhinal cortex, which plays a crucial role in episodic memory (Shimada et al., 2017). Significant individual differences exist in the relationship between episodic memory performance and sleep duration; specifically, poorer memory performance in older adults is strongly associated with shorter sleep duration (Hokett et al., 2021). On the other hand, episodic memory functions as more than mere information storage; it serves as the foundation for constructing self-awareness and emotion regulation strategies in middle-aged and older adults. When sleep duration is insufficient and causes emotional fluctuations, the functional robustness of episodic memory plays a pivotal moderating role. Studies suggest that optimizing sleep state may have a positive impact on episodic memory performance (Tassone et al., 2023). Furthermore, research demonstrates that the unconscious activation of episodic memory networks can predict psychological well-being (Philippe et al., 2012).

Executive function is a top-down, high-level cognitive mechanism that controls and coordinates lower-level psychological processes, playing a central role in self-regulation and goal-directed behavior (Snyder et al., 2015). On one hand, the pathway by which physical activity influences sleep duration extends beyond mere physiological fatigue; executive function plays a pivotal regulatory role within this process. Research indicates that diverse forms of physical activity can significantly enhance executive function (Montalva-Valenzuela et al., 2022). Short sleep duration or sleep disorders in older adults are often associated with structural atrophy and attenuated functional connectivity in the prefrontal cortex; however, physical activity enhances executive function by activating these prefrontal regions, thereby effectively regulating sleep duration (Daly et al., 2014). On the other hand, insufficient sleep duration in middle-aged and older adults leads to abnormally heightened reactivity in the subcortical limbic system, inducing psychological and emotional hyperarousal. Executive function, through its inhibitory control capabilities, effectively mitigates anxiety and depression via top-down regulation, thereby promoting mental health. Studies demonstrate that individuals with inadequate sleep duration also exhibit compromised executive function (Parrilla et al., 2025). Interventions that strengthen executive function can enhance the ability to self-regulate and inhibit negative thoughts, thereby increasing resilience and promoting psychological well-being (Shin and Brunton, 2024). Based on these theoretical underpinnings, we propose Hypothesis 5.

*Hypothesis 5*: The moderating effects of the cognitive function sub-dimension memory-executive exhibit differences.

**Figure 1:**
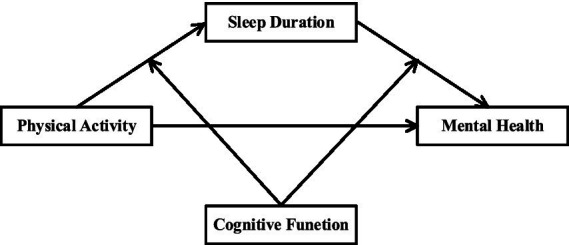
Theoretical model.

This study is based on exploring the mediating role of sleep duration and the moderating role of cognitive functioning from a physical activity perspective to inform solutions to the mental health problems of middle-aged and older adults, and Figure 1 illustrates the mediating moderating model used in this study.

## 2 Materials and methods

### 2.1 Participants and procedures

**Figure 2:**
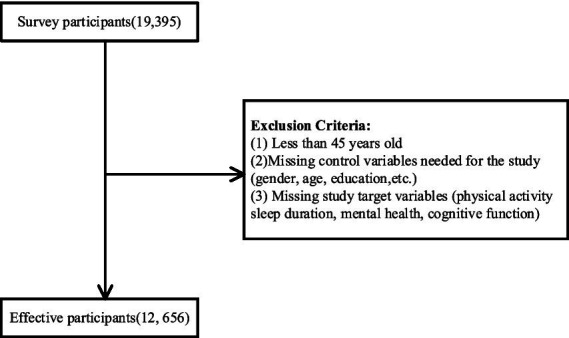
Sampling procedure table.

The data of this study are from the CHARLS database, which has been carrying out a national baseline survey since 2011, using the hierarchical sequential sampling method, probability sampling in proportion to the size of the population of the unit, with subjects covering more than 150 county-level units, more than 450 village-level units, about 10,000 households, and more than 17,000 person-times, which is a wide range of coverage, and a large base of subjects with a certain degree of representative and scientific (Zhao et al., 2014). In this study, the 2020 (fifth wave) data from this database were selected for investigation and research, and this study aimed to explore the role of physical activity on mental health, the mediating role of sleep duration, and the moderating role of cognitive function in middle-aged and elderly people. The exclusion criteria for the sample of this study: (1) age below 45 years; (2) missing covariates required for the study (gender, age, education level, place of residence, etc.); (3) missing variables targeted for the study (physical activity, sleep duration, mental health, and cognitive functioning). Ultimately, a total of 12,656 subjects were included. The sample specifics are shown in Table 1, and sample selection process is shown in Figure 2.

**Table 1:** Demographic information.

Variables	Item	Number	Percentage (%)
Gender	Male	6,567	51.9
	Female	6,089	48.1
Age	45–50 years	1,249	9.9
	51–55 years	2,498	19.7
	56–60 years	2,545	20.1
	61–65 years	2074	16.4
	Over 65 years	4,290	33.9
Level of education	No Formal Education (Illiterate)	3,688	29.1
	Did not Finish Primary School	3,254	25.7
	Elementary School	3,586	28.3
	Middle School and above	2,128	16.8
Marital status	Married (living together; not living together)	11,186	88.4
	Not in marriage (divorced; widowed; unmarried)	1,470	11.6
Current address	City/Town	3,701	29.2
	Rural	8,951	70.7
Chronic disease	0 species	2,558	20.2
	1 species and above	10,098	79.8

### 2.2 Variables measurement

#### 2.2.1 Physical activity

The CHARLS questionnaire measures physical activity, mainly using the International Physical Activity Ques-tionnaire (IPAQ), which categorizes the respondents’ physical activity time into time divided into 0 min, 10–29 min, 30–119 min, 120–239 min The median value was taken for calculation. The intensity of physical activity was categorized into: high intensity, such as climbing, running and farming activities; medium intensity. Such as brisk walking, playing tai chi, etc.; low intensity, such as walking. The metabolic equivalent (MET) was calculated with the knowledge of the duration and intensity of the subjects’ physical activity, and in calculating the metabolic equivalent, the value of high-intensity physical activity was assigned as 8.0, medium-intensity as 4.0, and low-intensity as 3.3, and the formula was: metabolic equivalen*t* = intensity of physical activity × number of days of physical activity × duration of daily physical activity (Lee et al., 2011). The physical activity scores were also converted to a range of 0–100 for analysis.

#### 2.2.2 Sleep duration

Sleep duration was derived from the China Family Health Survey, specifically the item “During Last Month Average Hours of Actual Sleep”. It is worth noting that although sleep duration is a foundational and widely used metric for sleep health, it may not comprehensively reflect the multidimensionality of sleep quality. Consequently, the findings regarding sleep duration are best interpreted within this specific conceptual context (Zhang et al., 2026).

#### 2.2.3 Mental health

The CHARLS questionnaire investigated the psychological aspects, mainly using the Depression Self-Rating Scale (CES-D), which responds to 8 items of negative psychological emotions, such as worrying about small things, inattention, low mood, tiredness, poor sleep, loneliness and difficulty in moving on with life, etc., and the present study assigns reverse values to such questions. There were also 2 positive psychological emotions, such as hopefulness for the future and feeling happy. The total score of the mental health section was 30 points, ranging from “seldom or not at all” to “most of the time” on a 4-point Likert scale, with values ranging from “0–3,” and the higher the score, the better the mental health of the subject. With higher scores indicating higher levels of mental health (Wu et al., 2023).

#### 2.2.4 Cognitive function

The CHARLS measures cognitive functioning in two main areas: episodic memory and executive function. Episodic memory tests the subjects’ ability for immediate and delayed recall, with scores ranging from 0 to 20; executive function is measured in three areas: orientation, calculation, and drawing, with scores ranging from 0 to 11. In order to more comprehensively reflect the subjects’ cognitive ability, this study follows previous studies by summing the episodic memory and executive function scores, resulting in a score range of 0 to 31. Higher scores indicate better cognitive functioning (Hou et al., 2024).

### 2.3 Statistical analysis

Data analysis for this study was performed using SPSS 27.0 software and the PROCESS macro. Following the completion of descriptive statistical analyses and correlation analyses, the research hypotheses were tested. First, after controlling for covariates, Model 4 of the PROCESS macro was used to examine the mediating role of sleep duration, and Model 58 of the PROCESS macro was employed to test the moderating role of cognitive function. Additionally, Model 58 of the PROCESS macro was utilized to examine the moderating effect of the cognitive function sub-dimension memory-executive. Finally, a cross-lagged model was constructed to strengthen causal inference.

## 3 Results

### 3.1 Descriptive statistics

In Table 2 the mean, standard deviation and correlation matrix of the variables are demonstrated. As shown in the table, there is a high correlation between the variables. Specifically, middle-aged and older married men who entered to live in the countryside, were younger, more educated, and did not have chronic diseases had higher levels of mental health and cognitive functioning. Differently, physical activity and sleep duration were better among middle-aged and older adults living in urban areas, and physical activity levels were generally higher among middle-aged and older adults with lower levels of education. Consistent with previous predictions, sleep duration, mental health, and cognitive functioning were positively correlated among middle-aged and older adults. Inconsistent with previous predictions, there was a negative relationship between physical activity and mental health and cognitive function in middle-aged and older adults, which may be due to inappropriate intensity of physical activity.

**Table 2:** Analysis of relevant results.

Variables	M	SD	1	2	3	4	5	6	7	8	9	10
Gender	0.52	0.5	1									
Marital status	0.88	0.32	0.11^**^	1								
Current address	0.71	0.46	0.01	0.04^**^	1							
Age	61.53	9.02	0.12^**^	−0.23^**^	−0.09^**^	1						
Level of education	2.33	1.07	0.18^**^	0.09^**^	−0.34^**^	−0.15^**^	1					
Chronic disease	0.8	0.40	−0.01	−0.04^**^	−0.02^*^	0.19^**^	−0.04^**^	1				
Physical activity	23.59	22.58	0.05^**^	0.083^**^	0.17^**^	−0.14^**^	−0.12^**^	−0.03^**^	1			
Sleep duration	6.10	1.73	0.11^**^	0.076^**^	0.03^**^	−0.07^**^	0.06^**^	−0.10^**^	0.03^**^	1		
Mental Health	21.97	6.21	0.16^**^	0.12^**^	−0.14^**^	−0.08^**^	0.22^**^	−0.17^**^	−0.05^**^	0.28^**^	1	
Cognitive function	12.82	3.22	0.06^**^	0.12^**^	−0.22^**^	−0.24^**^	0.45^**^	−0.06^**^	−0.08^**^	0.07^**^	0.28^**^	1

**p < 0.01; *p < 0.05.

### 3.2 Physical activity and mental health: a test of the mediating effect of sleep duration

This study employed Model 4 of the PROCESS macro for SPSS to examine the mediation effect after controlling for covariates. As shown in Table 3, physical activity positively predicted the mental health level of middle-aged and elderly people (*β* = 0.002, *t* = −6.757, *p* < 0.001), so the research Hypothesis 1 was verified. Moreover, physical activity can positively predict the sleep duration of middle-aged and elderly people (*β* = 0.001, *t* = 2.980, *p* < 0.01); at the same time, the sleep duration can also positively predict the mental health level of middle-aged and elderly people (*β* = 0.031, *t* = 32.825, *p* < 0.001), which indicates that the sleep duration plays a mediator role in the relationship between the physical activity and the mental health of middle-aged and elderly people, i.e., Hypothesis 2 was validated.

**Table 3:** Mediation model test.

Variant	Sleep duration				Mental health			
	*β*	*t*	LLCI	ULCI	*β*	*t*	LLCI	ULCI
PA	0.001	2.980^**^	0.001	0.003	0.002	−6.757^***^	−0.020	−0.011
SD					0.031	32.825^***^	0.944	1.064
*R* ^2^	0.001^**^				0.081 ^***^			

***p < 0.001; **p < 0.01. PA is physical activity; SD is sleep duration.

### 3.3 Physical activity and mental health: a test of the moderating effect of cognitive function

This study utilized Model 58 of the SPSS PROCESS macro to examine the moderated mediation effect after controlling for covariates. As presented in Table 4, physical activity positively predicted mental health in middle-aged and older adults (*β* = 0.002, *t* = −4.405, *p* < 0.001), supporting Hypothesis 1. Furthermore, physical activity exerted a significant positive predictive effect on sleep duration (*β* = 0.003, *t* = 3.733, *p* < 0.001), and sleep duration, in turn, positively predicted mental health (*β* = 0.054, *t* = 6.952, *p* < 0.001), thereby validating Hypothesis 2. Regarding moderation, cognitive function positively moderated the relationship between physical activity and sleep duration (*β* = 0.001, *t* = −2.910, *p* < 0.01). Similarly, cognitive function positively moderated the effect of sleep duration on mental health (*β* = 0.009, *t* = 2.236, *p* < 0.05). These findings support Hypotheses 3 and 4.

**Table 4:** Moderated mediation model tests.

Variant	Sleep duration				Mental health			
	*β*	*t*	LLCI	ULCI	*β*	*t*	LLCI	ULCI
PA	0.003	3.733^***^	0.005	0.015	0.002	−4.405^***^	−0.014	−0.006
CG	0.007	7.639^***^	0.039	0.066	0.107	6.603^***^	0.498	0.919
SD					0.054	6.952^***^	0.271	0.485
PA × CG	0.001	−2.910^**^	−0.002	−0.001				
SD × CG					0.009	2.236^*^	0.002	0.036
*R* ^2^	0.006^***^				0.146^***^			

***p < 0.001; **p < 0.01; *p < 0.05. PA is physical activity; CG is cognitive function; SD is sleep duration.

**Figure 3:**
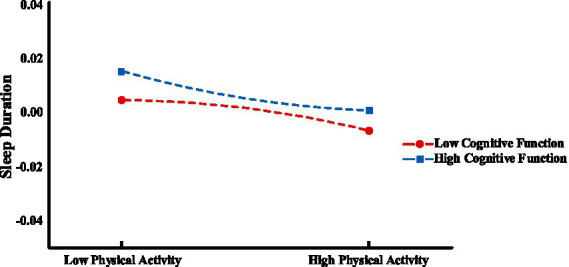
Moderating effects of cognitive functioning between physical activity and sleep duration.

**Figure 4:**
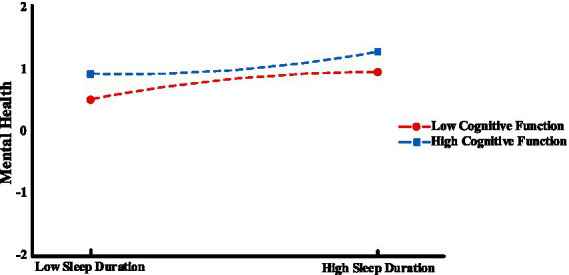
Moderating effects of cognitive functioning between sleep duration and mental health.

In order to reveal specific moderating effects, we categorized participants into “low” and “high” groups based on M ± 1SD and then performed a simple slope analysis. As shown in Figures 3, 4, the results indicated that when cognitive functioning was at a low level (M−1SD), there was a predictive effect of physical activity on sleep duration (*β* = 0.001, *t* = 4.633, *p* < 0.001), whereas when cognitive functioning was at a high level (M + 1SD), there was no predictive effect of physical activity on sleep duration (*β* = 0.001, *t* = 0.282, *p* > 0.05). This suggests that the predictive effect of physical activity on sleep duration decreases progressively as cognitive functioning increases. On the other hand, when cognitive functioning was at a lower level (M−1SD), there was a predictive effect of sleep duration on mental health (*β* = 0.036, *t* = 24.61, *p* < 0.001), and when cognitive functioning was at a higher level (M + 1SD), there was a stronger predictive effect of sleep duration on mental health (*β* = 0.046, *t* = 22.137, *p* < 0.001). This suggests that the predictive effect of sleep duration on mental health will gradually increase with cognitive functioning.

### 3.4 Physical activity and mental health: a test of the moderating effect of memory-executive

This study employed Model 58 of the PROCESS macro for SPSS to examine the moderating effects of the cognitive function sub-dimension memory-executive after controlling for other covariates. As shown in Tables 5, 6, memory did not significantly moderate the pathway from physical activity to sleep duration in middle-aged and older adults (*β* = 0.001, *t* = −1.475, *p* = 0.14), nor did it significantly moderate the pathway from sleep duration to mental health (*β* = 0.012, *t* = 0.681, *p* = 0.496). In contrast, executive exerted a significant positive moderating effect on the pathway from physical activity to sleep duration (*β* = 0.001, *t* = −2.624, *p* < 0.01) and a significant positive moderating effect on the pathway from sleep duration to mental health (*β* = 0.023, *t* = 1.755, *p* < 0.05). These findings indicate that memory-executive has a significant moderating effect in the model, a result that also validates Hypothesis 5 of this study.

**Table 5:** Moderated mediation model tests.

Variant	Sleep duration			Mental health		
	*β*	*t*	*P*	*β*	*t*	*p*
PA	0.001	2.334	0.02	0.001	−6.111	0.001
ME	0.055	4.428	0.001	0.642	5.751	0.001
SD				0.97	11.905	0.001
PA × ME	0.001	−1.475	0.14			
SD × ME				0.012	0.681	0.496
*R* ^2^	0.122^***^			0.002^***^		

***p < 0.001. PA is physical activity; SD is sleep duration; ME is memory.

**Table 6:** Moderated mediation model tests.

Variant	Sleep duration			Mental health		
	*β*	*t*	*P*	*β*	*t*	*p*
PA	0.001	3.397	***	0.001	−4.06	***
EX	0.065	6.415	***	0.498	5.969	***
SD				0.826	7.778	***
PA × EX	0.001	−2.624	**			
SD × EX				0.023	1.755	*
*R* ^2^	0.122^***^			0.002^***^		

***p < 0.001; **p < 0.01; *p < 0.05. PA is physical activity; SD is sleep duration; EX is executive.

### 3.5 Physical activity and mental health: a cross-lag model

**Figure 5:**
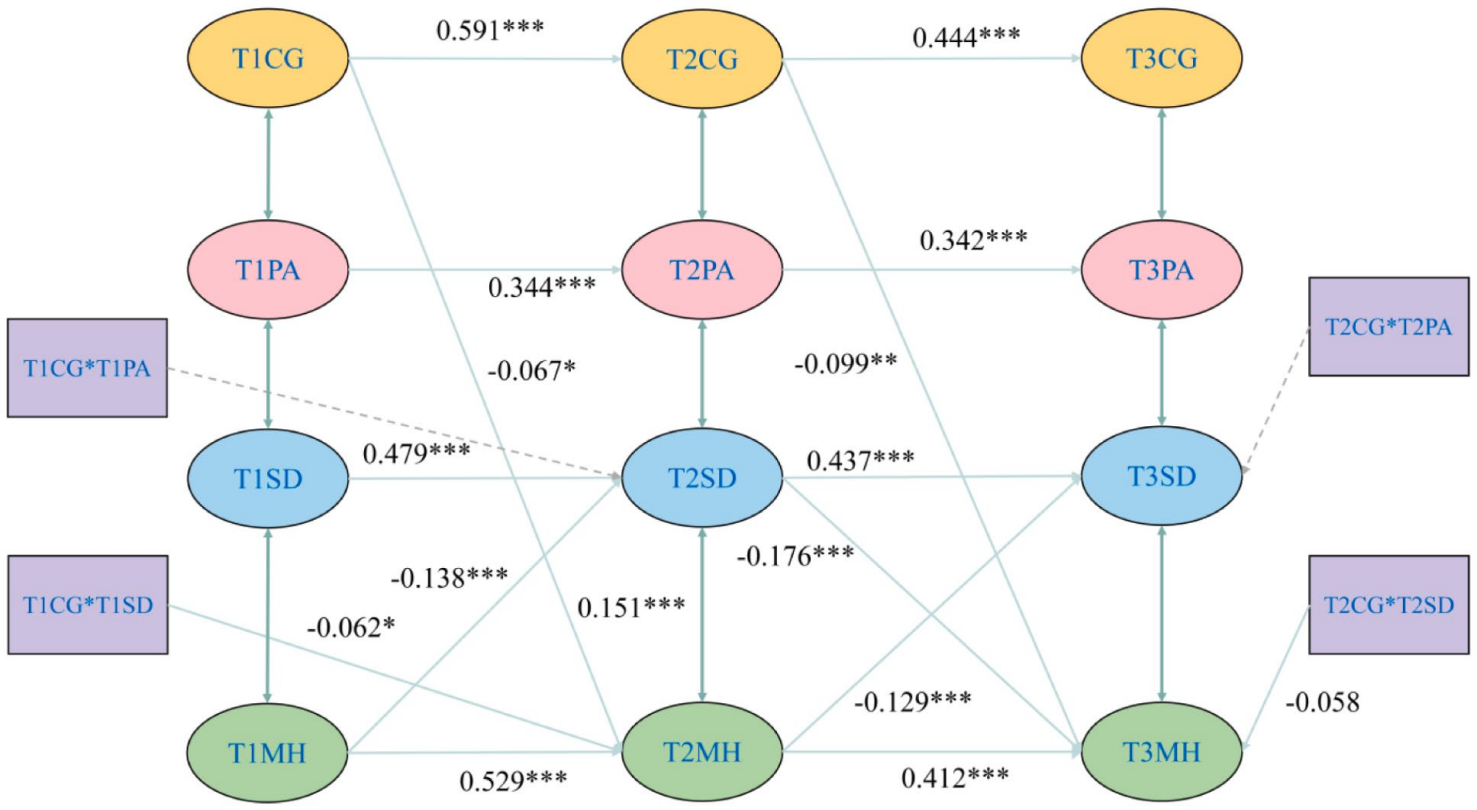
Cross-lag model diagram. ***p < 0.001; **p < 0.01; *p < 0.05. PA is physical activity; CG is cognitive function; SD is sleep duration; MH is mental health.

This section utilizes data from three waves of the CHARLS database (2015–2020). Following data screening, a total of 724 panel data entries were obtained. In this analysis, the mental health scores were not reverse-coded; therefore, higher numerical values indicate lower levels of mental health. We constructed interaction terms between the independent variable and the moderator at the previous time point (*T_n_*) to test the moderating role of the moderator (*T_n_*) on the effect of the independent variable (*T_n_*) on the mediator at the subsequent time point (*T*_*n* + 1_). Similarly, interaction terms between the mediator (*T_n_*) and the moderator (*T_n_*) were constructed to test the moderating role of the moderator (*T_n_*) on the effect of the mediator (*T_n_*) on the dependent variable (*T*_*n* + 1_). The results are presented in Figure 5. The model demonstrated a good fit: *χ*^2^ = 84.524, df = 36, CFI = 0.980, TLI = 0.950, SRMR = 0.026, RMSEA = 0.043.

Figure 5 displays all significant paths and their corresponding coefficients. For the sake of conciseness and readability, non-significant paths are not presented in the figure; detailed path coefficients are provided in Table 7. The cross-lagged path analysis indicated that Wave 1 cognitive function significantly and positively regulated Wave 2 mental health (*β* = −0.067, *t* = −2.036, *p* < 0.05), and Wave 2 cognitive function significantly and positively regulated Wave 3 mental health (*β* = −0.099, *t* = −3.003, *p* < 0.01). Furthermore, Wave 1 mental health significantly and positively predicted Wave 2 sleep duration (*β* = −0.138, *t* = −3.53, *p* < 0.001), and Wave 2 mental health significantly and positively predicted Wave 3 sleep duration (*β* = −0.129, *t* = −3.82, *p* < 0.001). Regarding the interaction effects, the interaction term between Wave 1 sleep duration and Wave 1 cognitive function significantly and positively predicted Wave 2 mental health (*β* = −0.062, *t* = −1.978, *p* < 0.05). The interaction term between Wave 2 sleep duration and Wave 2 cognitive function showed a marginally significant positive prediction for Wave 3 mental health (*β* = −0.058, *t* = −1.897, *p* = 0.058). These findings suggest that cognitive function played a certain positive moderating role in the relationship between sleep duration and mental health.

**Table 7:** Cross-lagged model test.

Outcome variable	Predictor variable	*β*	*SE*	*t*	*p*
MH2					
	MH1	0.529	0.035	15.169	***
	SD1	−0.057	0.036	−1.57	0.116
	PA1	0.008	0.03	0.249	0.803
	CG1	−0.067	0.033	−2.036	*
	SD*CG1	−0.062	0.031	−1.978	*
SD2					
	MH1	−0.138	0.039	−3.53	***
	SD1	0.479	0.037	12.879	***
	PA1	0.063	0.034	1.858	0.063
	CG1	−0.045	0.036	−1.22	0.222
	PA*CG1	−0.018	0.033	−0.554	0.579
PA2					
	MH1	−0.03	0.04	−0.749	0.454
	SD1	0.013	0.036	0.36	0.719
	PA1	0.344	0.042	8.265	***
	CG1	−0.073	0.039	−1.868	0.062
CG2					
	MH1	−0.045	0.032	−1.394	0.163
	SD1	−0.04	0.034	−1.165	0.244
	PA1	−0.075	0.029	−2.601	0.062
	CG1	0.591	0.031	18.887	***
MH3					
	MH2	0.412	0.039	10.666	***
	SD2	−0.176	0.031	−5.578	***
	PA2	−0.003	0.029	−0.099	0.921
	CG2	−0.099	0.033	−3.003	**
	SD*CG2	−0.058	0.031	−1.897	0.058
SD3					
	MH2	−0.129	0.034	−3.82	***
	SD2	0.437	0.041	10.776	***
	PA2	0.016	0.029	0.56	0.576
	CG2	−0.017	0.034	−0.493	0.622
	PA*CG2	−0.009	0.035	−0.254	0.8
PA3					
	MH2	0.016	0.036	0.433	0.665
	SD2	−0.013	0.035	−0.368	0.713
	PA2	0.342	0.042	8.215	***
	CG2	−0.038	0.035	−1.112	0.266
CG3					
	MH2	−0.034	0.03	−1.104	0.27
	SD2	0.042	0.03	1.393	0.164
	PA2	0.011	0.028	0.381	0.703
	CG2	0.444	0.036	12.413	***

***p < 0.001;**p < 0.01;*p < 0.05. PA is physical activity; CG is cognitive function; SD is sleep duration; MH is mental health.

Physical activity did not yield a significant beneficial effect in this longitudinal analysis. This outcome may be attributed to the demographic characteristics of the participants (middle-aged and older adults aged 45 and above) and the relatively extended follow-up period of the survey. This population typically exhibits a natural physiological decline in mental health, sleep duration, and cognitive function. While physical activity can mitigate these age-related changes, it may be insufficient to fully offset this downward trajectory over time.

Based on these longitudinal findings, we conclude that cognitive function serves as a significant positive predictor of mental health in middle-aged and older adults. Furthermore, mental health significantly predicts sleep duration, and cognitive function plays a positive moderating role in the relationship between sleep duration and mental health.

## 4 Discussion

This study investigated the effects of physical activity on the mental health of middle-aged and older adults, and examined the mediating role of sleep duration as well as the moderating role of cognitive function. Although previous studies have examined the effects of physical activity on mental health—for example, a study of 847 undergraduate students found that higher total physical activity was associated with better mental health (Rodríguez-Romo et al., 2022)—the roles of sleep duration and cognitive function within the mechanism linking physical activity to mental health remain insufficiently explored in related studies. Therefore, based on the relationship between physical activity and mental health, this study investigated the mediating role of sleep duration and the moderating role of cognitive function.

### 4.1 The impact of physical activity on mental health

The results of the study showed that physical activity showed a positive relationship with the mental health of middle-aged and older adults (*β* = 0.002, *t* = −6.757, *p* < 0.001), and it still showed a positive relationship with the mental health of middle-aged and older adults after cognitive function was introduced as a moderating variable (*β* = 0.002, *t* = −4.405, *p* < 0.001). In other words, within the appropriate range, as physical activity improves, the mental health of older adults also improves, which verifies Hypothesis 1 of the study, and also the present study is generally consistent with previous studies. For example, a study on the relationship between physical activity and mental health among adults in the U.S., which identified 7,674 respondents through a randomized sample across the country, found that there was a curvilinear relationship between physical activity and health, and that participation in physical activity was most effective in terms of benefits to an individual’s mental health within a threshold of 2.5–7.5 h per week (Kim et al., 2012). Furthermore, a survey of adolescent mental health in Northern Ireland found that not only was there a positive association between adolescents’ own physical activity and their mental health, but also the physical activity and mental health of their parents, which supports the need for family-based physical activity in the development of adolescent physical activity (Davidson et al., 2024). Research also indicates that physical activity alleviates depressive symptoms in middle-aged and older adults by regulating biomarkers such as brain-derived neurotrophic factor (BDNF), enhancing mitochondrial function, and promoting melatonin secretion (Zhang et al., 2025). In reality, middle-aged and elderly people are mostly in the free period after retirement, and due to the discomfort of being out of work and the lack of family companionship, they are prone to anxiety, loneliness and depression. In this case, by taking the initiative to participate in physical activities, it expands the social relationship and strengthens the health of the body, alleviates the negative emotions of the middle-aged and the elderly to a greater extent, and promotes the development of their psychological health.

### 4.2 Mediating effects of sleep duration

The results of this study indicate that physical activity not only directly predicts the mental health status of middle-aged and older adults but also indirectly affects it through sleep duration. Specifically, sleep duration plays a mediating role between physical activity and mental health (*β* = 0.001, *t* = 2.980, *p* < 0.01; *β* = 0.031, *t* = 32.825, *p* < 0.001). This implies that, within a normal range, as physical activity levels increase in this population, sleep duration improves concurrently, thereby promoting mental health both directly and indirectly. This conclusion validates Hypothesis 2 of the present study and aligns with previous findings. For instance, a meta-analysis on the impact of physical activity on sleep found that low-to-moderate intensity physical activity has a more significant effect on sleep outcomes, particularly in children and older adults (Zhao et al., 2023). Similarly, a study of rural residents in Ecuador revealed that the proportion of good sleepers was lower among those with lower physical activity levels, whereas it was higher among physically active individuals, suggesting a positive correlation between moderate physical activity and good sleep (Del Brutto et al., 2020). Furthermore, research indicates that as a non-pharmacological intervention, physical activity not only enhances physiological function in women but also helps mitigate the excessive secretion of stress hormones, thereby improving sleep architecture and reducing psychological anxiety (Li et al., 2025). By revealing the positive predictive effect of exercise on sleep duration in middle-aged and older adults, this study underscores the critical role of regular physical activity in promoting ‘healthy aging.

The association between sleep duration and mental health has also attracted significant attention from researchers. For instance, a study involving 17,639 Koreans found significant associations between abnormal sleep duration and stress levels, the experience of depressive symptoms, suicidal ideation, and psychiatric consultation behaviors (Lee et al., 2015). Meanwhile, a study conducted in Hefei, China, involving 5,743 individuals aged 45 and above, revealed that sleep duration in middle-aged and older adults is closely related to their mental health status. A noteworthy gender-specific difference observed was that the correlation between sleep duration and mental health was significantly stronger in middle-aged and older women compared to men in the same age group (Meng et al., 2025). Furthermore, in reality, middle-aged and older populations often face age-related issues such as insomnia, shortened sleep duration, and low sleep efficiency (Ouyang and Sun, 2019). This also leads to daytime fatigue, irritability, anxiety, and other mood fluctuations. Regular and moderate physical activity can regulate the circadian rhythms of middle-aged and older adults, promoting a more relaxed and fulfilled mental state, thereby effectively improving their sleep duration, alleviating negative emotions, and enhancing their mental health levels.

### 4.3 Moderating effects of cognitive functions

The results indicate that cognitive function plays a moderating role in the impact of physical activity on sleep duration among middle-aged and older adults (*β* = 0.001, *t* = −2.910, *p* < 0.01), and similarly moderates the impact of sleep duration on the mental health of this population (*β* = 0.009, *t* = 2.236, *p* < 0.05). In other words, among middle-aged and older adults with higher levels of cognitive function, the influence of physical activity on sleep duration is more significant, and the impact transmitted to mental health through sleep duration is also more pronounced—a conclusion that has been validated by prior research. For instance, a cross-sectional study indicated that for chronic stroke patients, the interaction between moderate-to-vigorous physical activity and sleep duration is significantly associated with cognitive function (Stein et al., 2025). Additionally, research suggests that physical activity can buffer against some of the detrimental effects of low sleep efficiency on cognitive function (Sewell et al., 2023). However, the present study found that cognitive function is not only regulated by physical activity and sleep duration but also effectively modulates the mechanistic pathway through which physical activity affects sleep duration. This finding further deepens the understanding of the mechanistic associations among cognitive function, physical activity, and sleep duration.

On the other hand, a study involving 4,744 older adults found that both sleep deprivation and excessive sleep are factors associated with an increased risk of depressive symptoms, whereas adequate sleep duration facilitates memory consolidation and brain repair, thereby enhancing individual cognitive function (Yang et al., 2025). Furthermore, a large-scale survey of 206,719 Korean adults revealed that individuals with abnormal sleep duration are more susceptible to cognitive decline; meanwhile, mental health issues such as depression and anxiety, along with unhealthy lifestyle behaviors like smoking, alcohol consumption, and sedentary behavior, are also high-risk factors for cognitive decline (Joo et al., 2021). A study focusing on older Chinese adults indicated that a sleep duration of 6 to 7 h constitutes the optimal window for enhancing overall cognitive function. Concurrently, sleep deprivation may be associated with depression, and both can exacerbate the risk of cognitive impairment (Tang et al., 2025). This study reveals the impact of sleep duration and mental health on adult cognitive function and further points out that cognitive function plays a moderating role in the relationship between sleep duration and mental health.

### 4.4 Moderating effects of memory-executive

The results indicate that memory and executive function play significant moderating roles in the model, with their moderating effects displaying distinct differences. Regarding the pathway through which physical activity influences sleep duration, physical activity does not automatically translate into high-quality sleep; rather, it requires adherence management and a regular physical activity plan. Middle-aged and older adults with robust executive function are better able to plan their exercise and inhibit maladaptive behaviors that disturb sleep. This self-regulation mechanism can significantly enhance cognitive function, thereby maximizing the sleep benefits derived from physical activity. In contrast, although episodic memory can provide beneficial contextual knowledge for exercise, mechanisms based merely on recall lack practical utility in guiding behavior; therefore, their moderating effect on the ‘exercise-sleep’ relationship may be weaker. For instance, research has shown a bidirectional association between physical activity and executive function, where impaired executive function leads to declined exercise participation (Daly et al., 2014). Further research indicates that sleep is a natural physiological regulatory process related to self-regulation and emotion; individuals with weaker executive function may be unable to effectively complete goal-directed tasks during the sleep decision-making process, thereby leading to sleep problems (Wang et al., 2026).

In the pathway whereby sleep duration affects mental health, abnormal sleep duration often serves as a precipitating factor for psychological issues such as anxiety and depression. Middle-aged and older adults with robust executive function can sufficiently activate regions related to the prefrontal cortex to implement top-down self-regulation, effectively inhibiting negative emotions triggered by physiological fatigue, thereby enhancing their mental health. Conversely, superior episodic memory alone does not equate to excellent emotional regulation ability; older adults with better episodic memory may recall painful experiences associated with insomnia more vividly, which effectively offsets potential regulatory benefits. This may result in the moderating effect of episodic memory being non-significant in the ‘sleep duration–mental health’ pathway. For instance, research indicates that optimal sleep duration is correlated with gray matter volume in multiple brain regions (such as the prefrontal cortex), whereas sleep problems impair executive function and affect psychological mood (Ansari Targhi and Mazandarani, 2024). Other studies suggest that executive function is crucial for decision-making and task processing but declines with age; improving sleep can optimize executive function and reduce the risk of dementia (Sen and Tai, 2023), while enhancing executive function has a positive impact on the mental health of middle-aged and older adults (Fusi et al., 2022).

### 4.5 Limitations and future recommendations

Despite being grounded in a solid theoretical foundation, this study is subject to several limitations: (1) by focusing solely on the mediating role of sleep duration and the moderating role of cognitive function, other potential variables influencing the association between physical activity and mental health may have been overlooked; (2) while sleep duration is a fundamental and widely used indicator of sleep health, it may not fully capture the multidimensional nature of sleep quality across different metrics; (3) as the sample was comprised exclusively of middle-aged and older adults, the generalizability of the findings to other age groups or specific populations requires further verification.

Future research should focus on the following directions: (1) integrating multidimensional variables—such as incorporating social support and inflammatory markers as potential mediators or moderators—would help to more precisely elucidate the diverse pathways through which physical activity influences mental health; (2) subsequent studies are advised to employ standardized multidimensional scales (e.g., the Pittsburgh Sleep Quality Index [PSQI]) to assess sleep quality more comprehensively; (3) future research should target different age and cultural groups to explore effective mechanisms for enhancing the mental health of diverse populations.

### 4.6 Research implications

This study contributes to the field of psychology by pioneering the exploration of the relationships among physical activity, sleep duration, cognitive function, and mental health in middle-aged and older adults. In terms of specific mechanisms, the research reveals the vital mediating role of sleep duration in the relationship between physical activity and mental health, confirming that physiological recovery processes (such as sleep) serve as a bridge for physical activity to improve mental health. This underscores the potential of non-pharmacological interventions in enhancing mental health. Simultaneously, this study identifies cognitive function as a crucial moderator, deepening the understanding of individual differences in physical and mental development. The results indicate that the level of cognitive function not only regulates the efficacy of physical activity in improving sleep duration but also moderates the extent to which sleep duration translates into mental health benefits. Furthermore, the study found that the cognitive function sub-domain memory-executive exerts a significant moderating effect within the model, and that the moderating roles of memory-executive exhibit distinct differences. Finally, to strengthen causal inference, a cross-lagged model was constructed. The results demonstrate that in a longitudinal cohort, cognitive function in middle-aged and older adults can significantly and positively influence mental health, while mental health can positively predict sleep duration; additionally, cognitive function plays a positive moderating role in the impact of sleep duration on mental health. In conclusion, these insights hold significant implications for strengthening physical activity, improving cognitive function, and enhancing sleep duration and mental health status in the elderly, laying a solid empirical foundation for developing health promotion strategies to address aging in the future.

## 5 Conclusion

The results of this study indicate that: (1) physical activity is positively correlated with mental health in middle-aged and older adults; (2) sleep duration mediates the relationship between physical activity and mental health; (3) cognitive function plays a moderating role in both stages of the mediation process; (4) the memory-executive subdomains exhibited differential moderating effects in middle-aged and older adults; (5) longitudinally, cognitive function positively predicted mental health, mental health predicted sleep duration, and cognitive function positively moderated the relationship between sleep duration and mental health.

Specifically, physical activity improves sleep duration and mental health status in middle-aged and older adults, and these effects are more pronounced in individuals with higher cognitive function. Therefore, to enhance physical activity levels and cognitive function, personalized activity plans should be formulated based on individual interests and appropriate exercise intensity to ensure safety and effectiveness. On this basis, it is crucial not only to encourage middle-aged and older adults to engage in group activities such as square dancing and Tai Chi to strengthen social interaction while boosting physical fitness for holistic health, but also to stimulate relevant brain regions through memory games and intellectual activities. This directly enhances cognitive function, ultimately forming a virtuous cycle where physical and mental health mutually reinforce each other.

## Data Availability

The raw data supporting the conclusions of this article will be made available by the authors, without undue reservation.
